# Retention in care among clinically stable antiretroviral therapy patients following a six‐monthly clinical consultation schedule: findings from a cohort study in rural Malawi

**DOI:** 10.1002/jia2.25207

**Published:** 2018-11-18

**Authors:** Alison Wringe, Caoimhe Cawley, Elisabeth Szumilin, Leon Salumu, Isabel Amoros Quiles, Estelle Pasquier, Charles Masiku, Sarala Nicholas

**Affiliations:** ^1^ Department of Population Health London School of Hygiene and Tropical Medicine London UK; ^2^ Independent consultant Berlin Germany; ^3^ Médecins sans Frontières Paris France; ^4^ Médecins sans Frontières Chiradzulu Malawi; ^5^ Epicentre Paris France

**Keywords:** differentiated care, Malawi, antiretroviral therapy, retention, cohort study

## Abstract

**Introduction:**

Longer intervals between clinic consultations for clinically stable antiretroviral therapy (ART) patients may improve retention in care and reduce facility workload. We assessed long‐term retention among clinically stable ART patients attending six‐monthly clinical consultations (SMCC) with three‐monthly fast‐track drug refills, and estimated the number of consultations “saved” by this model of ART delivery in rural Malawi.

**Methods:**

Stable patients (aged ≥18 years, on first‐line ART ≥12 months, CD4 count ≥300 cells/mL ^3^, without opportunistic infections, not pregnant/breastfeeding) were eligible for SMCC, with three‐monthly drug refills from community health workers. Early enrollees were those starting SMCC within six months of eligibility, while late enrollees started at least 6 months after first eligibility. Kaplan–Meier methods were used to calculate cumulative probabilities of retention, stratified by timing of their enrolment and from first six‐monthly clinical consultation. Cox regression was used to measure attrition hazards from the first six‐monthly clinical consultation and risk factors for attrition, accounting for the time‐varying nature of their eligibility and enrolment in this model of care.

**Results:**

From 2008 to 2015, 22,633 clinically stable patients from 11 facilities were eligible for SMCC for at least three months, contributing 74,264 person‐years of observation, and 18,363 persons (81%) initiated this model of care. The median time from eligibility to enrolment was 12 months and the median cumulative time on SMCC was 14.5 months. Five years after first SMCC eligibility, cumulative probabilities of retention were 85.5% (95% CI: 84.0% to 86.9%) among early enrollees and 93% (95% CI: 92.8% to 94.0%) among late enrollees. The cumulative probability of retention from first SMCC was 97.0% (95% CI: 96.7% to 97.3%) and 86% (95% CI: 85% to 87%) at one and five years respectively. Among eligible patients initiating SMCC, the adjusted hazards of attrition were 2.4 (95% CI: 2.0 to 2.8) times higher during periods of SMCC discontinuation compared to periods on SMCC. Male sex, younger age, more recent SMCC eligibility and WHO Stage 3/4 conditions in the past year were also independently associated with attrition from SMCC. Approximately 26,000 consultations were “saved” during 2014.

**Conclusion:**

After five years, retention among patients attending SMCC was high, especially among women and older patients, and its scale‐up could facilitate universal access to ART.

## 1 Introduction

In 2015, the World Health Organization (WHO) recommended immediate initiation of antiretroviral therapy (ART) for people living with HIV after their diagnosis, regardless of their immunological status 1, following evidence from randomized controlled trials and observational studies of its individual health benefits and prevention effects 2, 3. This approach holds great potential for reducing HIV‐related morbidity and mortality in sub‐Saharan Africa, if wide‐scale adoption of the WHO guidelines can be coupled with excellent rates of adherence to treatment 4.

Achieving these goals requires improvements in HIV testing and ART initiation rates, as well as in retention in care. In Malawi, substantial progress has been made towards achieving the The Joint United Nations Programme on HIV/AIDS (UNAIDS) 90‐90‐90 targets, with data from a household‐based national survey conducted in 2015 to 2016 indicating that 73% of people living with HIV aged 15 to 64 years reported knowing their HIV status, of whom 89% self‐reported current use of ART, and of whom 91% were virally suppressed 5. With self‐testing for HIV being scaled up in many countries including Malawi, HIV patient numbers are likely to further increase in the future, and may surpass health facility capacity, unless innovative models of ART delivery are widely adopted.

Differentiated models of HIV care have been advocated as a strategy for managing clinically stable patients by reducing the number of consultations in HIV clinics, which would free up health worker time to initiate new patients onto ART, and better address the needs of patients needing additional monitoring 6, 7, 8, 9, 10, 11. Furthermore, given that frequently cited barriers to appointment attendance include distance to health facilities, waiting times, competing responsibilities such as employment, and concerns over being seen 12, 13, 14, 15, patient‐centred models of care that addressed these issues may improve retention in care 7, 16.

Differentiated models of ART delivery can be broadly classified into individual models (facility or out‐of‐facility) and group models (health worker or client‐managed) 6. Several group and out‐of‐facility individual models have demonstrated promising results, including health worker‐led ART refill groups in South Africa 17, patient‐led community ART groups in Mozambique 18, 19, 20, 21 and community ART distributions points in the Democratic Republic of Congo 22. However, health worker‐managed or patient‐managed group strategies may require additional resources in order to scale‐up implementation, and concerns have been raised about their sustainability 23, 24. The facility‐based individual model should require fewer resources as it only involves appointment‐spacing of facility‐based clinical consultations and drug refills for stable patients 6. Appointment‐spacing allows clinically stable patients to attend HIV clinics less frequently for clinical assessments than the one or two‐monthly appointments required in many countries for ART patients, with drug refills provided between clinical consultations by lower cadre health workers 6, 25.

To date, there is a dearth of evidence from African settings on the effectiveness of facility‐based individual models of differentiated care, including data on long‐term retention rates and risk factors for attrition from care among patients enrolled in these models 26. The inclusion of differentiated ART delivery in the WHO guidelines since 2016 has been accompanied by calls to generate more evidence from demonstration projects of differentiated ART delivery on patient outcomes to facilitate their adoption within national HIV programmes, which will be key to achieving the UNAIDS 90‐90‐90 targets 6, 7, 8, 10, 26, 27. Analyses of routinely collected programme data, where facility‐based individual models have been introduced, can provide useful evidence on enrolment rates and long‐term treatment outcomes in programmatic settings, as well as insights into potential reductions in patient numbers within health facilities following its introduction. This paper describes long‐term retention in care, and risk factors for attrition from care among clinically stable ART patients who initiate six‐monthly clinical consultations (SMCC) with fast‐track three‐monthly refills from facility‐based community health workers in Chiradzulu District, Malawi over the period from 2008 to 2015, where Médecins sans Frontières (MSF) has been supporting the Ministry of Health's (MoH) HIV programme. We additionally estimate the number of clinic appointments “saved” as a result of its implementation since 2008.

## 2 Methods

### 2.1 Study setting

MSF has supported the Malawian MoH provision of HIV and tuberculosis services to around 270,000 persons in Chiradzulu district in southern Malawi since 1997 28. Free ART is available from the district hospital and 10 health centres. By 2013, HIV prevalence was 17%, and 77% of persons living with HIV had been diagnosed, 67% were on ART and 62% were virally suppressed 29.

### 2.2 The differentiated model of ART delivery

From January 2008, adult patients attending any HIV clinic in the district's health facilities were screened by a clinical officer, and those meeting eligibility criteria (see Table 1) were offered enrolment on the SMCC schedule consisting of health facility appointments for clinical assessments every six months, instead of every one or two months, and provision of a three‐month drug supply. Health surveillance assistants (HSA), who are paid community health workers, recruited and trained by the MoH and MSF, provided three‐monthly ART refills from each health centre or hospital pharmacy in between the SMCC, assessed adherence and monitored patients’ health and pregnancy status using a standardized assessment tool. Those on SMCC who were assessed by HSAs as having become no longer eligible (i.e. were pregnant, unwell or with adherence concerns) and patients choosing to opt‐out of SMCC (or wishing to seek medical care before their next scheduled SMCC) returned to their previous ART schedule at an HIV clinic of their choice and received clinical care as required. The main characteristics of the differentiated model of care, including eligibility criteria are summarized in Table 1 and further details can be found elsewhere 30.

**Table 1:** Key characteristics of the differentiated model of ART delivery and the previous standard model of ART delivery

	SMCC with fast‐track refills for eligible patients				ART delivery model prior to Jan 2008 and for non‐SMCC patients
	Jan 2008 to July 2013	Aug 2013 to Dec 2013	Jan 2014 to Dec 2014	Jan 2015 to July 2015	
Eligibility criteria					
Age	≥18 years	≥18 years	≥18 years	≥18 years	–
Time on first‐line ART	≥12 months	≥12 months	≥12 months	≥12 months, **or** ≥6 months **if** VL ≤ 1000 copies/mL	–
Most recent CD4 (cells/mL^3^) or VL (copies/mL)	CD4 ≥ 300	CD4 ≥ 300 or VL ≤ 1000	CD4 ≥ 200 or VL ≤ 1000	CD4 ≥ 200 or VL ≤ 1000	–
	All time periods:				
ART intolerance	No intolerance to ART				–
Opportunistic infections	No current tuberculosis or Kaposi's sarcoma within last year				–
Pregnancy status	Not pregnant, no children <2 years old				–
Who?					
Clinical consultation	Clinical officer				Clinical officer
ART refill	Health surveillance assistant (community health worker)				Clinical officer
When?					
Clinical consultation	Every six months				Every one to two months
ART refill	Every three months				Every one to two months
Monitor eligibility	Every three months				n/a
Where?	Health facility of patient's choice				Health facility of patient's choice

ART, antiretroviral therapy; SMCC, six‐monthly clinical consultations; VL, viral load.

### 2.3 Study design and data collection

We undertook a retrospective cohort analysis using data extracted from an electronic database where visit‐level data on patient records were routinely entered for programme monitoring purposes. Socio‐demographic characteristics were captured during the patient's first visit to the clinic, and clinical, immunological, pharmacy and adherence data were collected at follow‐up visits. Copies of follow‐up forms were forwarded from each health centre to a centralized location at the end of each day for data entry into an electronic database by trained clerks.

### 2.4 Data analysis

The study population included patients who were ever eligible for SMCC between January 2008 and July 2015. The baseline CD4 count and WHO stage measures were defined as the most recent results available up to one year prior to SMCC eligibility and one year prior to SMCC start. Pregnancy status was inferred from the number of weeks of amenorrhoea, and was used to define first and last possible pregnancy dates. Health facilities were defined as medium if they served a catchment population of approximately 15,000 to 25,000 persons, and as large if they served a population of over 25,000.

Kaplan–Meier probabilities for retention were calculated (i) after first eligibility date, stratified by enrolment status (never enrolled, early enrollee (within six months of first eligibility) or late enrollee (enrolled at least 6 months after first eligibility date)) and (ii) after first SMCC appointment. Attrition was defined as either reported death, or loss to follow‐up, with lost to follow‐up recorded for patients more than 60 days late for their last scheduled appointment. Cox proportional hazards regression was used to estimate crude and adjusted hazard ratios for attrition up to five years after first SMCC, and to identify risk factors for attrition. Eligibility and enrolment onto the SMCC schedule were considered as time‐dependent variables since patients could become ineligible for periods of time, for example if they became pregnant, but could later return to being eligible. Similarly, SMCC‐enrolled patients could leave and re‐enter the six‐monthly schedule, either due to changing eligibility status or for other reasons including a desire to return to shorter durations between appointments.

In five health centres, routine viral load monitoring was implemented from August 2013 in line with MoH protocols (at six and twenty‐four months after ART initiation and biannually thereafter). Among patients attending these health centres, the proportions with an undetectable viral load (defined as <1000 copies/mL) and the median time from SMCC initiation to viral load test were reported.

In order to calculate the total number of clinical appointments that were “saved” per year since SMCC introduction, we classified each patient visit as being either eligible or ineligible for SMCC. Eligible visits were further classified as: (a) a SMCC visit; (b) a drug refill visit with an HSA; or (c) routine visit (i.e. a non‐SMCC clinic visit among an SMCC‐eligible patient) and aggregated over a 12‐month period. The estimated number of appointments per category per year was then presented graphically. We defined the annual number of drug refill visits with an HSA as the annual number of clinical consultations that were saved. The total number of clinical appointments saved since the introduction of SMCC was calculated by summing these annual totals over the years of SMCC implementation.

### 2.5 Ethics

Ethical approval was provided by the National Health Sciences Research Committee in Malawi. As this is a secondary analysis of routinely collected programme data which were anonymized prior to analysis, consent for participation from patients was not sought. This research fulfils the exemption criteria set by the Médecins Sans Frontières Ethics Review Board for a posteriori analysis of routinely collected clinical data.

## 3 Results

### 3.1 Patient characteristics

In total, 47,904 patients aged 18 years and over were seen between January 2008 and July 2015, of whom 26,081 ever met the eligibility criteria for SMCC (Figure S1). The most common reasons for not being eligible for SMCC were having not yet initiated ART (n = 9615), having spent less than 12 months on ART (n = 7069) and having a baseline CD4 lower than the required threshold (n = 2282). Of those who were ever eligible for SMCC, 22,633 (87%) had at least three months of eligibility, and were included in the analyses, contributing 74,264 person‐years of observation.

Of the 22,633 individuals in the analysis (66% female), 19% never enrolled, 31% were early enrollees and 50% were late enrollees (Table 2). 18,363 (81%) of eligible patients initiated SMCC. At first SMCC, 66% of patients were women, and the majority were 25 years of age or older. Just over half were attending a medium‐sized health facility and 92% had a baseline CD4 count >300 cells/mm^3^, with a median duration since the CD4 count measurement of eight months. The median number of months from first eligibility for SMCC to starting the SMCC schedule was 12 months [interquartile range (IQR): 3 to 27 months].

**Table 2:** Baseline characteristics of (i) participants ever eligible for SMCC by enrolment category and (ii) participants at first SMCC

Variable	At first SMCC eligibility date			At first SMCC visit
	Never enrolled	Late enrollee	Early enrollee	All enrollees
	N = 4270	N = 11,240	N = 7123	N = 18,363
	n (%)	n (%)	n (%)	n (%)
Sex				
Male	1398 (33)	3508 (31)	2678 (38)	6186 (34)
Female	2872 (67)	7732 (69)	4445 (62)	12,177 (66)
Age				
18 to 24	306 (7)	368 (3)	259 (4)	420 (3)
25 to 34	1508 (35)	3388 (30)	2436 (34)	4945 (27)
35 to 44	1289 (30)	4262 (38)	2765 (39)	7206 (39)
≥45	1167 (27)	3222 (28)	1663 (23)	5792 (31)
Year of first SMCC eligibility				
2008 to 2009	1002 (23)	4137 (37)	863 (12)	n/a
2010 to 2011	792 (19)	3080 (27)	1088 (15)	n/a
2012 to 2013	1096 (26)	3399 (30)	2341 (33)	n/a
2014 to 2015	1380 (32)	624 (5)	2831 (40)	n/a
Size of health centre				
District hospital	940 (22)	2346 (21)	700 (10)	3046 (17)
Large health centre	1111 (26)	3238 (29)	2502 (35)	5740 (31)
Medium health centre	2179 (51)	5520 (49)	3921 (55)	9441 (51)
Other	40 (1)	136 (1)	0 (0)	136 (1)
Months since last CD4 result				
Median [IQR]	3 [1 to 8]	3 [1 to 6]	3 [1 to 6]	12 [3 to 26]
CD4 count in past year				
<300	516 (12)	357 (3)	727 (10)	1231 (7)
300 to 499	1822 (43)	5837 (52)	2945 (41)	6548 (36)
≥500	1334 (31)	4195 (37)	2701 (38)	8999 (49)
Missing	598 (14)	851 (8)	750 (11)	1585 (9)
WHO Stage 3/4 in past year				
Yes	2363 (55)	6105 (54)	2617 (37)	9189 (50)
No	1907 (45)	5135 (46)	4506 (63)	9174 (50)
Months since first clinic visit				
Median [IQR]	28 [15 to 50]	29 [16 to 49]	28 [16 to 48]	48 [29 to 72]
Months since ART start				
Median [IQR]	19 [12 to 35]	18 [12 to 35]	15 [12 to 28]	35 [23 to 60]
Months since first SMCC eligibility				
Median [IQR]	n/a	n/a	n/a	12 [3 to 27]

SMCC, six‐monthly clinical consultations; ART, antiretroviral therapy; IQR, interquartile range.Statistical significant differences were observed for all variables comparing never enrolled, late enrollee and early enrollee (Pearson chi‐square, *p* < 0.05).

### 3.2 Median cumulative time spent in states of SMCC enrolment and eligibility

**Figure 1:**
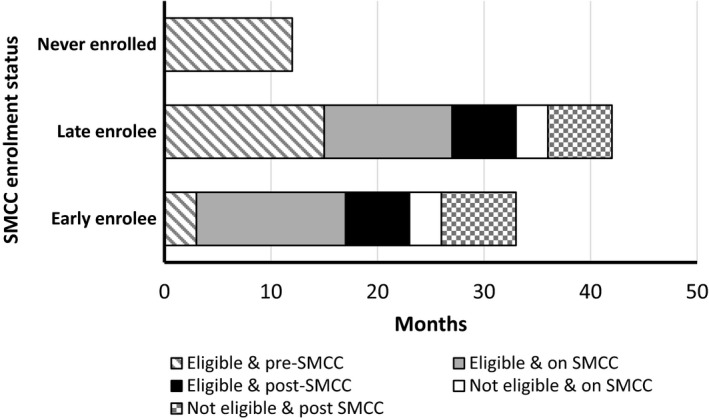
Cumulative median time spent in different states of SMCC eligibility and enrolment, stratified by enrolment category

Following first eligibility for SMCC, patients who never initiated the SMCC schedule spent a median cumulative time of 12 months being eligible for SMCC and 10 months being no longer eligible for SMCC (Figure 1). Among late enrollees, these durations prior to starting SMCC were broadly similar. The cumulative median total time spent eligible and on SMCC for late enrollees was only 12 months, while a further median of six months was spent being no longer on SMCC regardless of eligibility. Among early enrollees, as expected, time spent being eligible but not yet started on SMCC, or no longer eligible and not yet started on SMCC, was much shorter at approximately three months each. As with late enrollees, the cumulative median total time spent eligible and on SMCC among early enrollees was only just over one year, while a further median of six months was spent being no longer on SMCC regardless of eligibility. Interruptions to SMCC eligibility were less frequent among patients who enrolled early onto SMCC (16.0% compared to 36.2% among those never enrolled and 38.0% among late enrollee (*p* < 0.001, data not shown)).

### 3.3 Probability of retention by SMCC enrolment category and from first SMCC

**Figure 2:**
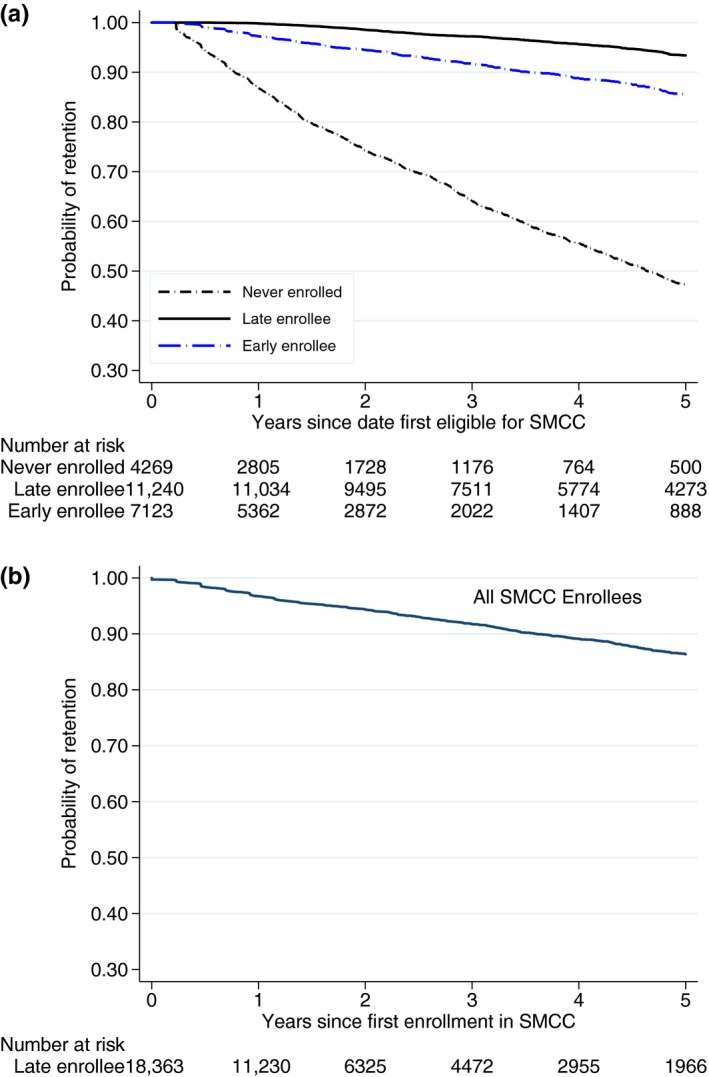
**(a)** Cumulative probability of retention in HIV care among patients ever eligible for the SMCC schedule by enrolment category. **(b)** Cumulative probability of retention in HIV care among patients from first SMCC consultation.

The median time retained in care after first SMCC eligibility was 3.2 years [IQR: 1.6 to 5.0]. The cumulative probability of retention in care one year after first SMCC eligibility was 86.8% (95% CI: 85.6% to 87.8%) among those who never enrolled, 97.3% (95% CI: 96.8% to 97.6%) among early SMCC enrollees and 99.8% (95% CI: 99.7% to 99.9%) among late SMCC enrollees, while the corresponding figures at five years were 47.4% (95% CI: 45.0% to 49.7%), 85.5% (95% CI: 84.0% to 86.9%) and 93.4% (95% CI: 92.8% to 94.0%) respectively (Figure 2a). Among all patients who ever initiated SMCC, the median time retained in care was 1.2 years [IQR: 0.7 to 3.0]. The cumulative probability of retention at one year was 97.0% (95% CI: 96.7% to 97.3%) and at five years was 86% (95% CI: 85% to 87%) (Figure 2b).

### 3.4 Crude and adjusted hazard ratios for attrition

Among those who ever started on the SMCC schedule, most of the follow‐up time was spent being both eligible and enrolled on SMCC, during which the rate of attrition was 26.4/1000 person‐years (Table 3). The crude hazards ratio for attrition was 2.3 (95% CI: 2.0 to 2. 8) in the period when patients were eligible but off SMCC and 2.3 (95% CI: 1.8 to 2.9) in the periods when patients were no longer eligible and off SMCC, when compared with periods when patients were both eligible for SMCC and following the SMCC schedule. In the unadjusted Cox models, the hazard of attrition was also higher in men, those aged 18 to 24 years and 25 to 34 years, those more recently eligible for SMCC, those with no recorded CD4 counts in the year prior to initiating SMCC, those with shorter durations on ART and those with WHO Stage 3/4 conditions in the past year.

**Table 3:** Rates, unadjusted, and adjusted hazard ratios from Cox regression for attrition among participants who ever started SMCC

All patients (N = 18,363)	Attritions	Person‐years (py)	Attrition rate/1000 py	Unadjusted Cox model		Adjusted Cox model^a^	
				cHR (95% CI)	*p*‐value	aHR (95% CI)	*p*‐value
SMCC periods							
Eligible and off SMCC	188	3584	52.5	2.3 (2.0 to 2.8)	<0.01	2.6 (2.2 to 3.1)	<0.01
Eligible and on SMCC	751	28,466	26.4	1.0		1.0	
Not eligible and off SMCC	87	1713	50.8	2.3 (1.8 to 2.9)	<0.01	2.4 (1.9 to 3.0)	<0.01
Not eligible and on SMCC	25	903	27.7	1.1 (0.7 to 1.6)	0.7	1.2 (0.8 to 1.8)	0.36
Sex							
Male	404	11,750	37.9	1.2 (1.1 to 1.4)	<0.01	1.3 (1.2 to 1.5)	<0.01
Female	647	22,916	30.5	1.0		1.0	
Age at baseline							
18 to 24	54	778	53.1	2.6 (2.0 to 3.5)	<0.01	2.6 (1.9 to 3.4)	<0.01
25 to 34	347	10,102	34.4	1.3 (1.1 to 1.5)	<0.01	1.3 (1.1 to 1.5)	<0.01
35 to 44	368	13,899	26.5	1.0		1.0	
≥45	282	25,380	28.5	1.1 (0.9 to 1.3)	0.41	1.1 (0.9 to 1.2)	0.44
Size of health centre							
District hospital	152	4500	33.8	1.0 (0.9 to 1.2)	0.91	1.0 (0.9 to 1.3)	0.5
Large health centre	292	11,547	25.3	0.8 (0.7 to 0.9)	<0.01	0.8 (0.7 to 0.9)	<0.01
Medium health centre	601	18213	33.0	1.0		1.0	
Other	6	405	14.8	0.5 (0.2 to 1.0)	0.05	0.6 (0.2 to 1.2)	0.15
Year of first SMCC eligibility							
2008 to 2009	353	15,796	22.3	1.0		1.0	
2010 to 2011	310	9535	32.5	1.5 (1.3 to 1.8)	<0.01	1.7 (1.4 to 2.0)	<0.01
2012 to 2013	300	7298	41.1	2.1 (1.7 to 2.4)	<0.01	2.4 (2.0 to 2.9)	<0.01
2014 to 2015	89	2037	43.7	2.2 (1.7 to 2.8)	<0.01	2.3 (1.8 to 3.1)	<0.01
Last pre‐SMCC CD4^b^							
<300	36	984	50.7	1.2 (0.8 to 1.6)	0.41	0.9 (0.7 to 1.4)	0.78
300 to 499	420	13,807	33.5	1.0			
≥500	523	18,544	30.7	0.9 (0.8 to 1.1)	0.24	1.0 (0.9 to 1.2)	0.89
Missing	72	133	68.2	1.7 (1.3 to 2.2)	<0.01	1.7 (1.3 to 2.2)	<0.01
Month since ART start							
<24 months	386	12,337	31.3	1.2 (1.0 to 1.4)	0.05	0.9 (0.7 to 1.2)	0.33
24 to 47 months	457	15,799	28.9	1.1 (1.0 to 1.3)	0.20	1.1 (0.9 to 1.4)	0.5
≥48 months	316	11974	26.4	1.0		1.0	
Month since first visit							
<24 months	261	7685	34.0	1.2 (1.1 to 1.4)	0.04	1.1 (0.8 to 1.4)	0.49
24 to 47 months	429	16,011	26.8	1.0 (0.8 to 1.1)	0.64	0.8 (0.7 to 1.0)	0.05
≥48 months	469	16,413	28.6	1.0		1.0	
Cumulative WHO Stage 3/4^b^							
Recorded	572	18,140	31.6	1.1 (1.0 to 1.2)	0.17	1.2 (1.0 to 1.3)	0.02
Not recorded	479	16,526	29.0	1.0		1.0	

SMCC, six‐monthly clinical consultations; ART, antiretroviral therapy, cHR, crude hazard ratio; aHR, adjusted hazard ratio.

^a^Adjusted for all variables in the table; ^b^past year.

In the adjusted models, patients had 2.6 times higher hazards of attrition (95% CI: 2.2 to 3.1) during periods when patients were eligible but no longer on SMCC, and 2.4 (95% CI: 1.9 to 3.0) times higher hazards of attrition during periods when patients were not eligible and off SMCC, compared to periods when they were both eligible and enrolled on SMCC There was no difference in adjusted hazards of attrition among patients during periods when they were not eligible and on SMCC compared to periods when they were eligible and on SMCC (adjusted hazard ratio (aHR) = 1.2; 95% CI: 0.8 to 1.8). In the adjusted models, there was an increased hazard of attrition among patients aged 18 to 24 years (aHR 2.6, 95% CI: 1.9 to 3.4) and in those aged 25 to 34 years (aHR 1.3, 95% CI: 1.1 to 1.5) compared to patients aged 35 to 44 years, and among men compared to women (aHR 1.3, 95% CI: 1.2 to 1.5). There was a trend in increased attrition hazards with more recent calendar years of SMCC eligibility (*p* < 0.01). Patients with recorded WHO Stage 3/4 conditions in the past year had higher attrition hazards compared to those who did not (aHR 1.2, 95% CI: 1.0 to 1. 3).

### 3.5 Virological outcomes

In total, 4649 patients who were ever eligible for SMCC first enrolled on the SMCC schedule at one of five health centres between August 2013 and July 2015 where routine viral load testing was available. Of these patients, at least one viral load measurement was available for 1742 (38%) individuals. Of these, 93% (n = 1619) of viral load measurements were <1000 copies/mL. The viral load test was done a median of 21 months following first SMCC enrolment [IQR: 14 to 28].

### 3.6 Appointment savings

**Figure 3:**
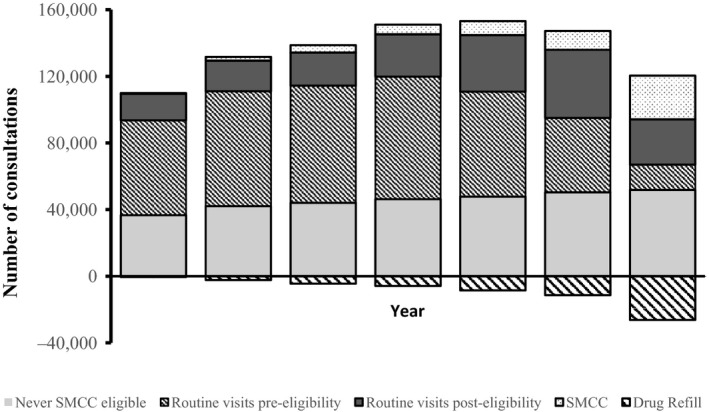
Estimated annual number of clinic appointments by appointment type

In 2008, when the strategy was first introduced, a total of 110,389 clinic visits were recorded across the 11 facilities among patients ≥18 years of age (Figure 3). As the strategy was rolled out, the number of routine visits among patients who never became eligible for differentiated ART (including those not yet on ART) increased steadily from under 37,000 per year to approximately 52,000 by 2014. The number of visits made by patients who were eligible for SMCC at that particular visit, but who had not yet enrolled on SMCC, peaked at just under 41,000 in 2013, and represents appointments that could have been saved if the programme had been able to initiate all eligible patients onto the SMCC schedule. The number of SMCC visits per year increased from <500 in 2008 to just over 26,000 during 2014, and corresponds to the increase in the number of three‐monthly drug refill visits made by SMCC patients to the HSAs that is the number of clinic visits “saved” as a result of SMCC implementation. Over the period from 2008 to 2014, the total number of visits “saved” equates to just short of 62,000. The annual total number of visits in the health facilities only starts to decline in 2013 from a peak of 161,619 due to the gradual rollout of SMCC.

## 4 Discussion

Our results indicate that SMCC with three‐monthly ART refills from community health workers represents a feasible and effective strategy for delivering first‐line ART in this rural African district. The cumulative probability of retention was high among both early and late SMCC enrollees following their eligibility for SMCC, and also high among all patients following SMCC initiation with 97% and 86% retained after one and five years respectively of follow‐up. These retention rates compare very favourably with those previously observed among adults on first‐line ART in this setting, as well as elsewhere 31, 32, and are also broadly in line with those reported from studies reporting on other differentiated models of care 17, 19, 33.

Although the hazards of attrition were low among eligible patients who initiated SMCC, the adjusted hazards of attrition were 2.6 (95% CI: 2.2 to 3.1) times higher during periods of SMCC discontinuation compared to periods on SMCC, suggesting the need for careful patient monitoring among patients who discontinue SMCC. As with traditional models of ART delivery in many settings 34, men and young adults aged 18 to 24 years of age had higher attrition risks, indicating that differentiated models of care may not be sufficient to overcome age‐ and sex‐specific barriers to optimal ART outcomes, and that men and young people may require additional support or alternative differentiated care models to remain engaged with HIV care and treatment. Attrition risks were also lower for earlier patient cohorts, reflecting the longer average time spent being eligible for SMCC before enrolment, and the accompanying selection effects that meant that they were well‐established patients with excellent adherence histories by the time that they initiated SMCC.

Although 81% of all eligible patients were initiated on to SMCC, there was a median time of 12 months from first eligibility to first SMCC. Furthermore, the cumulative median time on SMCC was relatively short at 14 months among early and late enrollees, suggesting that efforts are needed to reduce the delay between becoming eligible for SMCC and moving to this schedule. Although further research is needed to understand and address patient and health systems barriers to SMCC uptake, it is likely that further health worker training on application of the SMCC protocols and ensuring patient readiness may reduce delays in SMCC initiation following eligibility.

This model of differentiated ART delivery in this setting saved just under 62,000 clinical consultations over seven years, despite the slow pace of the rollout. Assuming that a typical clinical officer conducts around 500 consultations per month, the total savings equates to almost 118 clinician‐months or approximately 10 clinician‐years for the health facilities in this district over the study period. During 2014, approximately 26,000 appointments had been moved outside the clinic during the year as a result of SMCC, corresponding to approximately sixty clinician‐months or five clinician‐years, and representing a substantial saving in health worker time in a country with severe workforce shortages.

These findings demonstrate that SMCC can play a key role in enabling ART programmes to continue their expansion as test and treat policies are introduced, by freeing up health worker time to initiate new patients on ART, and manage complicated cases such as patients with suspected treatment failure, opportunistic infections, or those taking second‐line regimens. Furthermore, this model of care is likely to be scalable in Malawi, and elsewhere, because the strategy requires fewer formally trained health workers and no additional resources than the current standard of care.

The findings from our analysis are consistent with studies investigating the effectiveness of community‐based or group‐based ART delivery strategies for clinically stable patients in other settings 9, 35 and emerging findings from studies on individual, facility‐based individual models 33, 36. Facility‐based individual models based on appointment‐spacing may be easier to implement, and thus possibly more likely to be scaled up by national HIV programmes. Many African countries explicitly recommend differentiated models of HIV care including appointment‐spacing 27, 37, in line with recent guidelines from WHO and the International AIDS Society that recommend differentiated models of HIV care for clinically stable ART patients 6, 10, 11.

There are various limitations that need to be considered when interpreting these findings, including those inherent to using routinely collected programmatic data such as missing information that could influence classifications of SMCC eligibility, or result in residual confounding (e.g. factors relating to marital status or partner use of HIV services). In particular, better documentation of reasons for delayed initiation and non‐initiation of SMCC among apparently eligible patients would help to understand the poorer outcomes among this group which precluded us from using them as a comparison group in our analyses, due to selection biases that this would introduce 26. Documentation of reasons for leaving the SMCC schedule, despite remaining eligible, would help assess reverse causality, and aid interpretation of the relatively high attrition hazards during these post‐SMCC periods. Furthermore, we were not able to measure mortality as an outcome, as the true status of patients who are lost to follow‐up from the clinic are not ascertained, and will thus include deaths, defaulters and undocumented transfers to other clinics. Nevertheless, as patients in the programme are given a copy of their records, and are permitted to attend other HIV clinics in the district for ART consultations, we expect that the number of undocumented transfers between clinics should be low, and thus attrition is likely to closely align to mortality. A further limitation was our inability to investigate virological outcomes among all SMCC patients since routine viral load testing was only introduced progressively in the district from 2013. Although the preliminary findings among the patients with viral load results are promising, future analyses should include viral load as a primary outcome measure to better ascertain the implications of SMCC on adherence, and this will become increasingly possible as viral load monitoring becomes more widespread.

Previous studies reporting retention rates among patients receiving ART through other models of differentiated care have had shorter follow‐up periods 18, 33, 35. The strength of this study was the ability to document retention over a five‐year follow‐up period as well as the possibility of including all HIV clinics within one district, resulting in a large cohort of patients under observation.

While these findings provide a much‐needed and widely called for contribution to the evidence base on differentiated models of ART delivery 7, 10, 27, our results also highlight several priorities for further research. This includes identifying the optimal time at which appointment‐spacing should start for first‐line ART patients and documentation of discontinuation rates, reasons and associated risk factors. Furthermore, qualitative studies are needed to elicit provider and patient perspectives on this model of care, including underlying reasons for its success, and to better understand why some SMCC‐eligible patients opt for the standard model of ART delivery. Such research could facilitate greater engagement of patients in further refining the SMCC model to best suit their needs, address their concerns and enhance its performance.

## 5 Conclusion

In conclusion, our findings demonstrate that SMCC with fast‐track refills for clinically stable, first‐line ART patients represents an effective and feasible strategy for delivering treatment, enabling substantial savings in terms of health worker input, providing more flexible access to care and achieving excellent outcomes under routine programme conditions. Further scale‐up of facility‐based consultation‐spacing strategies with three‐monthly fast‐track ART refills from community health workers should be encouraged, accompanied by ongoing programme monitoring and evaluation in other settings, as an important step towards achieving universal access to ART and a future free of AIDS.

## Competing interests

None to declare.

## Authors’ contributions

AW, SN and CC designed the study; CM, CC and SN oversaw the data management; SN, CC and AW designed or undertook the analysis; AW, SN, CC, ES, LS and EP interpreted the data; AW and CC wrote the first draft of the manuscript. All authors provided comments which were incorporated into subsequent drafts by AW.

## Supporting information


**Figure S1. **Flow chart showing inclusion and exclusion for the analysis.
