# Supplementary material for: Retention in care among clinically stable antiretroviral therapy patients following a six‐monthly clinical consultation schedule: findings from a cohort study in rural Malawi
Source: J Int AIDS Soc. 2018 Nov 18;21(11):e25207. doi: 10.1002/jia2.25207 (PMC6240757; doi:10.1002/jia2.25207)
Supplement: Supplementary file 1 — Figure S1. Flow chart showing inclusion and exclusion for the analysis. [file JIA2-21-e25207-s001.pptx]

## Slide 1
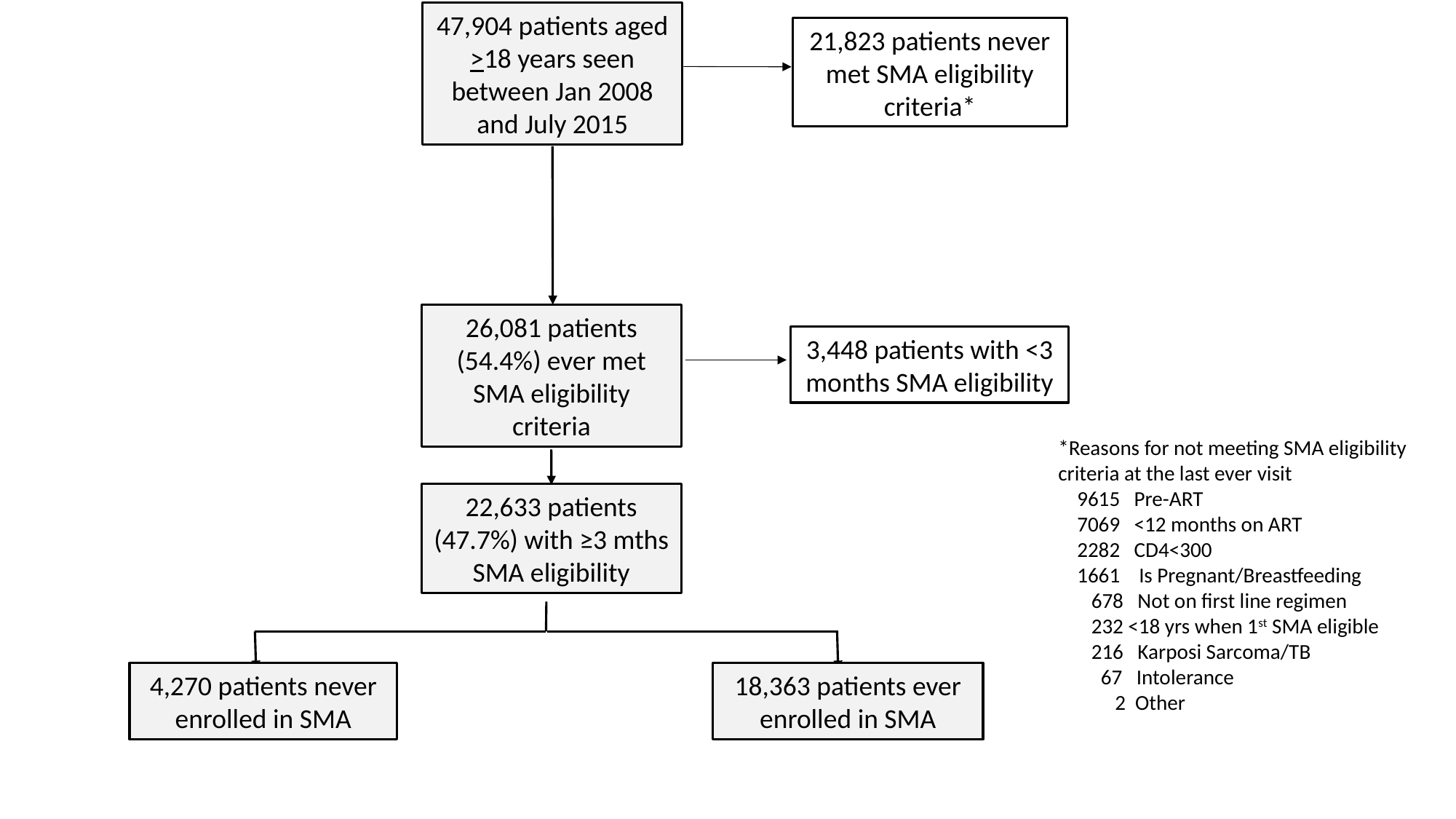

47,904 patients aged >18 years seen between Jan 2008 and July 2015
21,823 patients never met SMA eligibility criteria*
26,081 patients (54.4%) ever met SMA eligibility criteria
3,448 patients with <3 months SMA eligibility
22,633 patients (47.7%) with ≥3 mths SMA eligibility
4,270 patients never enrolled in SMA
18,363 patients ever enrolled in SMA
*Reasons for not meeting SMA eligibility criteria at the last ever visit
 9615 Pre-ART
 7069 <12 months on ART
 2282 CD4<300
 1661 Is Pregnant/Breastfeeding
 678 Not on first line regimen
 232 <18 yrs when 1st SMA eligible
 216 Karposi Sarcoma/TB
 67 Intolerance
 2 Other
